# A Multi-Sensor Fusion and CWT-CNN-BiLSTM-Based Approach for Small-Sample Fault Diagnosis in Rotating Machinery

**DOI:** 10.3390/s26144553

**Published:** 2026-07-17

**Authors:** Zhe Li, Zhangwen Zhou, Zhuojian Wang, Qi Chen

**Affiliations:** Aeronautics Engineering School, Air Force Engineering University, Xi’an 710038, China; fspm1321@163.com (Z.L.); uzwsyu@163.com (Z.Z.); zhuojianw1974@sina.com (Z.W.)

**Keywords:** rotating machinery, fault diagnosis, multi-sensor fusion, CWT, CNN-BiLSTM, small sample

## Abstract

Rotating machinery has been widely used in industries, but it often faces a high incidence of sudden failures under harsh operating conditions. Therefore, ensuring its safety and reliability is of utmost importance. However, fault diagnosis frequently encounters challenges such as limited training samples and the susceptibility of individual vibration sensors to external interference and noise. To enhance the recognition accuracy of rotating machinery under noisy and small-sample conditions, a fault diagnosis method for small samples based on multi-sensor fusion and CWT-CNN-BiLSTM is proposed in the paper. Firstly, the data from the multi-sensor is concatenated and fused, and then converted into a two-dimensional feature image through CWT. This study introduces a CNN-BiLSTM model designed to extract pivotal features from images. The feasibility of this method has been verified using the rotating machinery fault diagnosis database made available by the Korean Institute of Science and Technology. The average diagnostic accuracy achieved is 99.90%. The experimental results show that the proposed method results in more accurate and robust fault classification under small-sample conditions.

## 1. Introduction

Amid the ongoing advances in industrial technology, mechanical equipment is gradually moving towards integration and automation. The reliable operation of equipment has received unprecedented attention. As an important component of mechanical equipment, rotating machinery has been broadly adopted in aerospace, numerically controlled machine tools and wind turbines [1,2]. However, prolonged operation inevitably leads to failures or damage in key components. It will not only affect the performance of the entire system, but may also lead to equipment shutdown and cause greater economic losses [3,4]. Therefore, developing a fault diagnosis system for rotating machinery systems has important practical significance.

With the advancement of sensor and signal processing technologies, the vibration, acoustic, temperature, pressure and current signals for the condition monitoring of rotating machinery have been studied and realized [5,6,7,8]. But most of these traditional methods rely on manual feature extraction, which requires professional knowledge and diagnostic experience in the field of signal processing. The algorithms are often designed for specific types of faults or equipment, and they are sensitive and difficult to be generalized.

In recent years, diagnostic methods utilizing deep learning have become prevalent in the industry, primarily because they eliminate the need for manual feature engineering. A diverse array of models, including long short-term memory networks (LSTMs) [9], convolutional neural networks (CNNs) [10,11,12,13], deep autoencoders (DAEs) [14], gate recurrent units (GRUs) [15], deep belief networks (DBNs) [16], and bidirectional temporal convolutional networks (BiTCNs) [17] have been successfully applied to various industrial diagnostic tasks. At the same time, large language models (LLMs) are also used for fault diagnosis [18]. However, these methods typically demand substantial training data to achieve optimal performance [19]. In real-world engineering scenarios, critical equipment is rarely permitted to operate under fault conditions, limiting the availability of fault data. This scarcity can result in overfitting, particularly for deep learning models trained on small datasets, compromising their reliability [20]. Moreover, the characteristics of fault data can vary significantly under different operating conditions, further diminishing the generalization capabilities of these models. Consequently, their applicability may be severely restricted in certain scenarios. To address these challenges, it is crucial to develop fault diagnosis methods that are effective with limited fault data or small sample sizes, ensuring robust and reliable performance across diverse industrial applications.

Focusing on the small-sample problem, Xue proposed an innovative solution, the core of which lay in a multi-scale convolutional neural network integrated with a self-calibrated coordinate attention mechanism [21]. By utilizing dynamic models and transfer learning, Dong proposed a cutting-edge intelligent framework for fault diagnosis in rolling element bearings [22]. Similarly, meta-learning with hierarchical recurrent networks for data reconstruction has been proposed to enable accurate fault diagnosis for bearings with limited samples across varying conditions [23]. The conditional generative adversarial network model and graph convolutional network model have also been used for small-sample fault diagnosis [24,25,26]. However, these methods mainly rely on data from one vibration sensor for fault diagnosis, which is prone to external interference and noise. Environmental factors and operational fluctuations can significantly impact measurement accuracy, potentially leading to erroneous fault classification results.

To overcome the limitations of single-sensor approaches in fault diagnosis, researchers have increasingly turned to multi-sensor fusion techniques [27,28]. Multi-sensor fusion can be categorized into three main levels, data fusion, feature fusion, and decision fusion. Data layer fusion involves combining raw data from various sensors into a single and unified dataset, thereby providing a more comprehensive view of the system’s state. The feature layer fusion involves fusing the features extracted from different sensor signals by multiple feature extractors. The decision layer fusion strategy operates by synthesizing the independent diagnostic outcomes from multiple sensors, thereby enhancing the process’s robustness and reliability. Such fusion approaches have demonstrated their efficacy across a wide range of applications. For example, Guan developed a weighted strategy based on the kurtosis criterion to fuse three-dimensional sensor data into a single dataset, achieving improved diagnostic performance [29]. Zhu concatenated four sensor signals into a synthesized signal and learned the new signal through a deep Q-network, demonstrating excellent diagnostic accuracy [30]. Tong first applied fuzzy rank calculation to determine the decision score for each sensor, then fused these scores to reduce the gap between actual and predicted outcomes [31]. An enhanced voting strategy has been proposed by Shao, which achieved more accurate and reliable gearbox fault diagnosis by integrating the diagnostic results from several basic models [32]. Wang implemented a three-stage fusion method to achieve multi-level integration of vibration and torque signal features [33]. The residual convolutional fusion network was designed for multi-sensor bearing fault diagnosis by Ye [34]. Qiu combined image fusion with deep learning to develop a diagnosis method for rolling bearing fault [35]. However, most existing methods primarily rely on cascading and summation operations for feature fusion, which may not fully exploit the valuable information provided by all sensor signals. In addition, when the fault signals are obscured by strong noise or the sample size is insufficient, the performance of feature learning declines sharply. This will eventually lead to unsatisfactory diagnostic accuracy.

To enhance the recognition accuracy of rotating machinery under noisy and small-sample conditions, a fault diagnosis method for a small sample based on multi-sensor fusion and CWT-CNN-BiLSTM is proposed in the paper. Firstly, we selected and integrated sensor signals that can reflect various faults in rotating machinery, including vibration, control current, and temperature. The data is then transformed into two-dimensional images using CWT, so that the wavelet coefficients can encode the interactions among multiple physical fields. Next, a CNN-BiLSTM network model is designed to extract pivotal features from images. To address the problem of overfitting that occurs in model algorithms when the amount of sample data is small, and to improve their generalization ability, dropout layers are applied after both the pooling layers and the fully connected layers. The proposed method results in more accurate and robust fault classification under small-sample conditions.

An overview of the paper’s structure is given below. Section 2 covers the theoretical foundations of the fault classification model that integrates image fusion of multi-sensor and deep learning. The specifics of the proposed CWT-CNN-BiLSTM model are presented in Section 3. Section 4 details the experimental design and discusses the results. The study is concluded with key findings in Section 5.

## 2. Materials and Methods

The core theoretical underpinnings of the three primary technologies used in this study—CWT, CNN and BiLSTM—are detailed in this section. By innovatively integrating and optimizing these methodologies, a foundational framework is established for the subsequent innovation of methods.

### 2.1. CWT

The CWT stands as a classic time-frequency analysis technique, utilizing a scale-variable mother wavelet to scrutinize non-stationary signals, such as vibration signals. Through the conversion of a 1D time-domain signal into a 2D time-frequency diagram, CWT effectively reveals the localized energy distribution of signals across both time and frequency. This effectively overcomes the limitations of Short-Time Fourier Transform (STFT), which cannot simultaneously achieve time and frequency resolution due to the use of fixed window lengths. It also compensates for the drawback of traditional Fourier analysis, which entirely fails to provide temporal information [36]. CWT is particularly adept at capturing time-varying frequency components and transient shocks in non-stationary signals. It can offer high-resolution time-frequency characterization for fault diagnosis.

The principle is that the mother wavelet is translated on the time axis and scaled by the scale factor to generate a series of sub-wavelet functions. The signal’s time-frequency representation is obtained through the inner product calculation between the signal and the sub-wavelets, which yields the coefficient distribution across the time-frequency plane.

The essence of CWT lies in the mother wavelet Ψ(*t*). It is a function with localized and oscillatory properties. By scaling and translating the mother wavelet, a series of sub-wavelets are generated.

(1) $$\Psi_{ab}(t)=\frac{1}{\sqrt{a}}\Psi (\frac{t-b}{a})$$

where *a* represents the scale factor, *b* represents the translation factor, *a* ∈ *R*, *b* ∈ *R*, and *a* > 0.

The scale factor is introduced to realize the adaptive adjustment of the analysis window. When the scale factor is a small value, the wavelet function is compressed and the time resolution is high, which is suitable for analyzing the high frequency transient components. When the scale factor is a large value, the wavelet function is stretched and the frequency resolution is high, which is suitable for extracting the low frequency slow change features.

The CWT of the input *x*(*t*) is obtained by convolving this signal with a series of sub-wavelets and calculating the wavelet coefficients.

(2) $$W(a,b)=\frac{1}{\sqrt{a}}\int_{-\infty }^{\infty }x(t)\Psi^{*}(\frac{t-b}{a})dt$$

where W(a,b) denotes the wavelet coefficient, the scale factor a is inversely proportional to frequency, the translation factor b corresponds to a position on the time axis, and $\Psi^{*}(t)$ is the conjugate of the mother wavelet. The amplitude of the wavelet coefficient represents the energy distribution of a signal at specific times and frequencies.

The performance of the wavelet transform hinges on the selection of wavelet basis functions, with common choices including Haar, Coiflet, Morlet and Cmor wavelets. As the complex form of the Morlet wavelet, the Cmor wavelet exhibits stronger time-frequency-focusing characteristics and adaptive capabilities, making it a preferred choice for the CWT. By using MATLAB R2023b software to perform CWT processing on the acquired timing signals, two-dimensional time-frequency images can be generated for the subsequent analysis.

### 2.2. CNN

Being a prominent member of the deep learning family, CNN is a class of feedforward neural network that incorporates convolutional computations and deep architecture [37]. Specialized in processing visual data, CNN has demonstrated remarkable achievements in image processing and computer vision. Their robust learning capacity stems from the continuous transformation and extraction of underlying features, capturing unique signal characteristics across multiple levels. This process ultimately yields highly abstract distributed features, unveiling the fundamental patterns hidden within the original signals.

The standard CNN is generally composed of an input layer, several convolutional and pooling layers, and then passed through a fully connected layer and a softmax layer. It also includes some network optimization methods like dropout, Adam optimizer and batch normalization.

The input layer typically receives one-dimensional raw signals or two-dimensional images, then forwards them to subsequent convolutional and pooling layers aimed at feature extraction.

Through convolution operations, the convolutional layer captures features from specific regions of input images by applying convolution operations using kernels to feature vectors from the preceding layer. The results are then transformed nonlinearly via an activation function, producing the layer’s feature map output. Owing to its weight-sharing property, the convolutional layer reduces the number of network parameters and enhances the training efficiency. Its mathematical formulation is as follows:

(3) $$x_{j}^{l}=f(\sum_{i\in M_{j}}(x_{i}^{l-1}⊗k_{ij}^{l})+b_{j}^{l})$$

where ***M****_j_* denotes the quantity of the feature maps, ***k*** denotes the convolution kernel, ***b*** denotes the bias, $x_{j}^{l}$ denotes the output of the layer *l*, $x_{i}^{l}$ represents the input signal, *f* denotes the activation function, and ⊗ is the convolution operation.

CNN models commonly employ activation functions such as Tanh, Sigmoid and ReLU. The function form of ReLU is relatively simple. When the input is non-negative, the output matches the input; for negative inputs, the output is zero. By zeroing out some neurons, ReLU enhances network sparsity and reduces parameter interdependence, effectively mitigating overfitting. This makes it well-suited for training deeper neural networks, as it facilitates rapid parameter updates. Therefore, ReLU is typically chosen as the nonlinear activation function in CNN. The formal expression for ReLU is as follows.

(4) $$a_{i}^{l+1}(j)=f(y_{i}^{l+1}(j))=\max\left\{0,y_{i}^{l+1}(j)\right\}$$

where $y_{i}^{l+1}(j)$ is the convolution operation output, and $a_{i}^{l+1}(j)$ denotes the activation value of $y_{i}^{l+1}(j)$.

Following the convolutional layer, the pooling layer serves to downsample feature maps and decreases the number of trainable parameters, mitigating overfitting and enhancing the network’s translation invariance to input data. Standard pooling methods are average and maximum pooling. As the most widely used method, maximum pooling identifies the peak value within the pooling window, effectively retaining the most prominent features in the feature map. Its mathematical expression is as follows:

(5) $$P_{i}^{l+1}(j)=\max(q_{i}^{l}(t))$$

where $P_{i}^{l+1}(j)$ denotes the (*l* + 1)-th layer neuron output, $q_{i}^{l}(t)$ represents the value of the neuron *t* located in the *i*-th feature vector of layer *l*, t∈[(j−1)W+1,jW], and *W* denotes the size of the pooling area.

The fully connected layer bridges the final pooling layer and the output layer, with each node connected to all nodes in the preceding layer. It transforms the output features from the last pooling layer into a one-dimensional feature vector, enabling hierarchical feature aggregation. This vector is then mapped to the classification space, facilitating accurate predictions.

The output layer follows the fully connected layer to handle classification tasks, typically employing the Softmax function. This function transforms the neurons from the final fully connected layer into a probability distribution summing to 1, enabling accurate prediction of input data categories. The expression is as follows.

(6) $$soft\;\max(z_{j})=\frac{\exp(z_{j})}{\sum_{j=1}^{N}\exp(z_{j})}$$

where $z_{j}$ is the fully connected layer output.

### 2.3. BiLSTM

**Figure 1:**
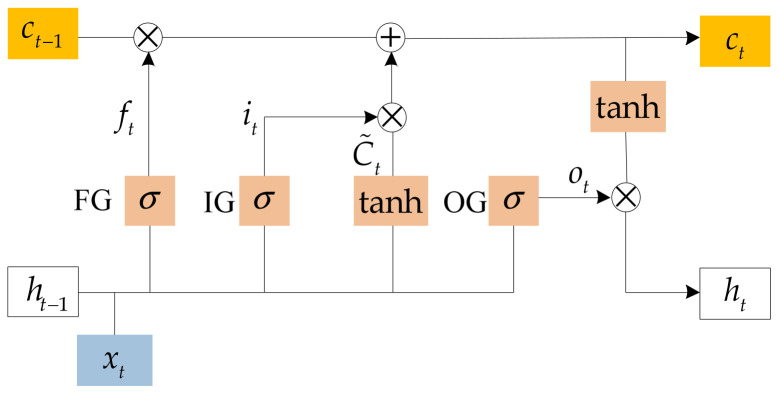
The structure of LSTM.

Designed to address the vanishing and exploding gradient problems in long sequences, LSTM constitutes a more sophisticated type of recurrent neural network. It achieves this through a gating mechanism that selectively retains or discards information. The core memory cell of LSTM contains three gates: the forget gate, input gate and output gate. Its network structure is shown in Figure 1. These gates ensure that information can selectively pass through, while the state of the transmission unit is only obtained through some linear operations, thus ensuring the invariance of information during transmission. Through the selective memory information, most of the information in the sample data is used to learn the deep features, and then to perform fault diagnosis.

The sample data undergoes the following three steps in LSTM. Firstly, the sample data passes through the forget gate to determine which information needs to be retained and which needs to be forgotten. Then, it passes through the input gate to update the cell state and identify the updated information. Finally, the information flows through the output gate to produce the network’s output. The corresponding mathematical formulation is provided below.

(7) $$\left\{\begin{array}{l} f_{t}=\sigma (W_{f}\cdot (h_{t-1},x_{t})+b_{f})\\ i_{t}=\sigma (W_{i}\cdot (h_{t-1},x_{t})+b_{i})\\ {\tilde{C}}_{t}=\tanh(W_{C}\cdot (h_{t-1},x_{t})+b_{C})\\ o_{t}=\sigma (W_{o}\cdot (h_{t-1},x_{t})+b_{o})\\ C_{t}=f_{t}\cdot C_{t-1}+i_{t}\cdot {\tilde{C}}_{t} \end{array}\right.$$

where $f_{t}$ denotes the forget gate, $i_{t}$ represents the input gate, $o_{t}$ is the output gate, ${\tilde{C}}_{t}$ is the input state at the current time step, $C_{t}$ is the internal state at the current time step, and σ is the activation function.

**Figure 2:**
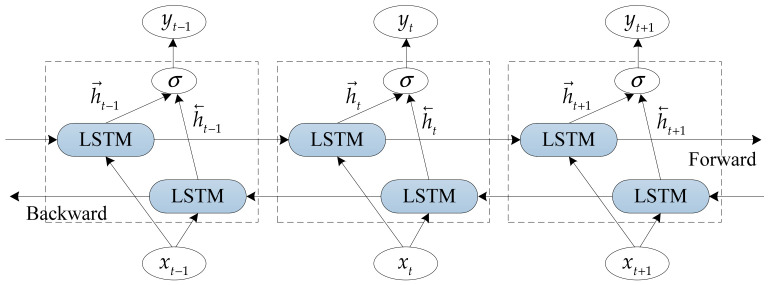
The structure of BiLSTM.

Although LSTM has memory capabilities to learn historical information, it can only process forward information and cannot effectively utilize backward information. The BiLSTM model improves upon LSTM by effectively addressing this limitation, thereby enhancing learning capacity. As depicted in Figure 2, BiLSTM processes input sample signals through both forward and backward LSTMs. The hidden layer receives input values computed from both directions, which are combined to determine the final value. This structure allows BiLSTM to capture context from both past and future information, improving overall performance. The formula for this integration is as follows:

(8) $$\left\{\begin{array}{l} {\vec{h}}_{t}=LSTM(x_{t},{\vec{h}}_{t-1})\\ {\overset{\leftarrow }{h}}_{t}=LSTM(x_{t},{\overset{\leftarrow }{h}}_{t-1})\\ y_{t}=\sigma (W_{t}\cdot ({\vec{h}}_{t},{\overset{\leftarrow }{h}}_{t})+b_{t}) \end{array}\right.$$

where ${\vec{h}}_{t}$ is the forward LSTM layer output, ${\overset{\leftarrow }{h}}_{t}$ is the backward LSTM layer output, and $y_{t}$ is the hidden layer output after BiLSTM stacking.

## 3. Multi-Sensor Fusion Fault Diagnosis Model Based on CWT-CNN-BiLSTM

**Figure 3:**
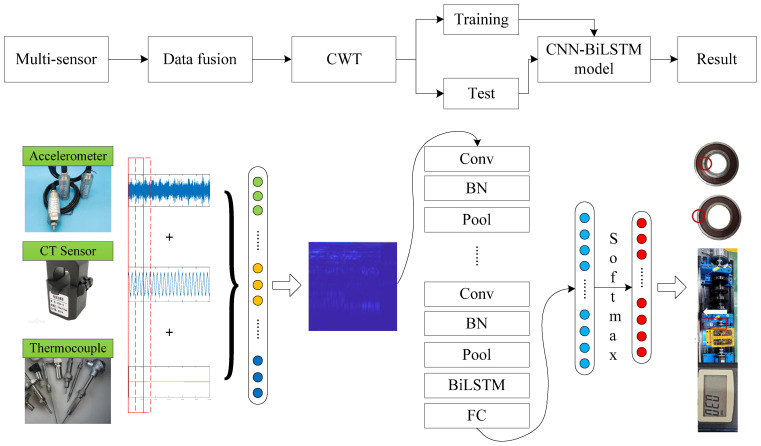
The structure of the fault diagnosis model.

The paper proposes a state recognition method based on multi-sensor fusion and CWT-CNN-BiLSTM. The structure of the fault diagnosis model is illustrated in Figure 3. The specific steps of the method are as follows:

Step 1: Deploy vibration, current, and temperature sensors on the mechanical rotating equipment to acquire different types of signal information.

Step 2: Apply a data window shifting technique to synchronously segment the raw multi-sensor signals with overlapping samples. The data from each sensor is divided into N segments of one-dimensional initial data samples.

Step 3: Concatenate and merge the synchronously processed one-dimensional initial data samples from multiple sensors into a single data sample, ultimately obtaining N concatenated data samples.

Step 4: Perform Continuous Wavelet Transform (CWT) on each concatenated data sample to generate a two-dimensional time-frequency image. After adjusting it to an appropriate size, save it to the sample set.

Step 5: Randomly split the two-dimensional time-frequency images into training and test sets at a specified ratio.

Step 6: Construct the CNN-BiLSTM model and initialize the network hyperparameters.

Step 7: Feed the training set in batches into the CNN-BiLSTM network for training, and use an optimization algorithm to iteratively optimize the model parameters.

Step 8: Input the test samples into the trained network for evaluation. Finally, the Softmax layer outputs the classification results and the test accuracy.

## 4. Experiment and Analysis of Fault Diagnosis

### 4.1. The Data Collected from Experiments

**Figure 4:**
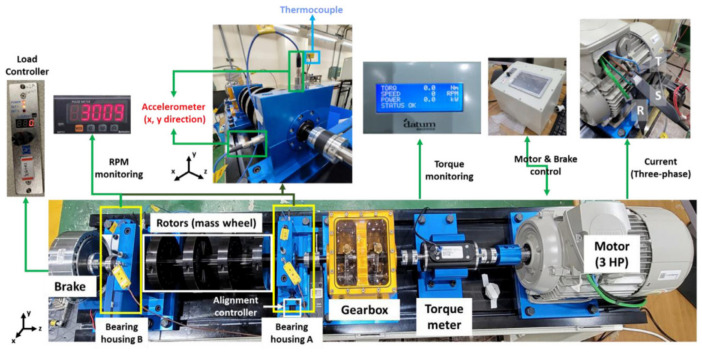
Configuration of the rotating machine testbed.

To assess the proposed algorithm’s performance, experiments were performed using samples from the Open Rotating Machinery Fault Diagnosis Database of Korea Advanced Institute of Science and Technology. The test rig (see Figure 4) comprises several key components: a SIEMENS motor (4-pole, 3 HP; Siemens AG, Munich, Germany), a hysteresis brake, a torque meter, a gearbox, rotors and bearing housing A and bearing housing B. The three-phase induction motor was supplied at 380 V and 60 Hz. Load was applied via the hysteresis brake (Valid Magnetic Ltd. AHB-3A; Fo Tan, Hong Kong, China) and measured by the torque meter (Datum Electronics M425; Datum Electronics Ltd., East Cowes, UK), with simulated loads of 0 Nm, 2 Nm and 4 Nm. Accelerometers (PCB35234; PCB Piezotronics, Inc., Depew, NY, USA) were mounted on bearing housings A and B to measure vibrations in both the x- and y-directions, following the ISO 10816-1:1995 [38] vibration installation guide. Additionally, two K-type thermocouples were fitted in each bearing housing for temperature measurement. Three Hioki CT6700 CT sensors (Hioki E.E. CORPORATION, Ueda, Japan) were installed to measure the motor current on each of the three phases (R, S, T). Data acquisition was performed at 3010 rpm to prevent spectral overlap with the 60 Hz line frequency. Three types of data—vibration, temperature and current—were acquired under the three load conditions, using a 25.6 kHz sampling frequency. For the experiment, samples were selected from ten distinct conditions under 0 Nm load for analysis: inner race faults (0.3 mm, 1.0 mm, 3.0 mm), outer race faults (0.3 mm, 1.0 mm, 3.0 mm), misalignment faults (0.1 mm, 0.3 mm, 0.5 mm) and the healthy bearing state [39].

### 4.2. Create Data Samples

**Figure 5:**
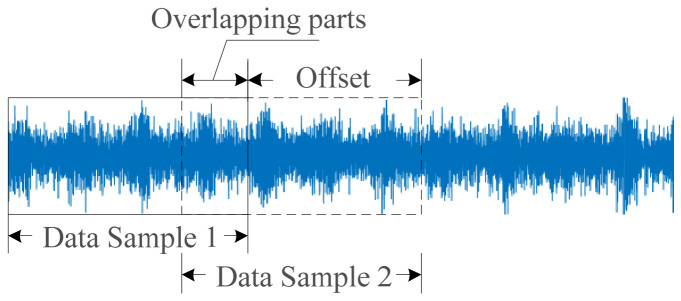
Sample segmentation using the data window shifting technique.

The data window shifting technique was employed to perform overlapping sample segmentation for data augmentation, which effectively preserves the continuity and periodicity inherent in time-series signals. Compared to non-overlapping segmentation, this overlapping strategy retains more of the correlation between adjacent elements. Consequently, the model can learn more robust features for classification. The segmentation process is illustrated in Figure 5.

The original signals under different fault conditions were segmented with a specified overlap ratio η to build the training and test sets. Assuming the length of a signal under a certain fault condition is N and the sample length is set to L, the sample expansion with overlap rate η is implemented as follows:

(1) The maximum number of segments obtainable from the current signal length is calculated as:
  (9) $$m=\left\lfloor \frac{N-L}{L\times \eta }\right\rfloor$$

where $\left\lfloor \cdot \right\rfloor$ denotes the floor function.

(2) Obtain each segmented sample. The location of the *i*-th sample in the original signal is given by:
  (10) $$x_{i}=signal[(i-1)\times L\times \eta +1:(i-1)\times L\times \eta +len],i\in [1,m]$$

where *x_i_* denotes the segmented data sample, and the overlap ratio *η* can be set to any value to meet varying requirements for sample size in different experimental or model training scenarios.

Regarding the selection of sample segment length, a shorter segment length can reduce training time and accelerate model convergence, but it may also result in less information contained within each sample. This could lead to the loss of partial information during subsequent nonlinear feature extraction, thereby affecting recognition accuracy. Conversely, a longer segment length increases the time required for algorithm convergence, which may compromise the real-time performance of the fault detection model. Therefore, selecting an appropriate sample length is crucial for balancing high fault recognition accuracy with fast convergence speed. Based on the sampling rate, data segments were extracted with a fixed length of 1024 points, and each subsequent segment starts 512 points after the previous one, generating a sample sequence with a 50% overlap rate. This ensures comprehensive coverage of fault characteristics along the temporal dimension.

**Figure 6:**
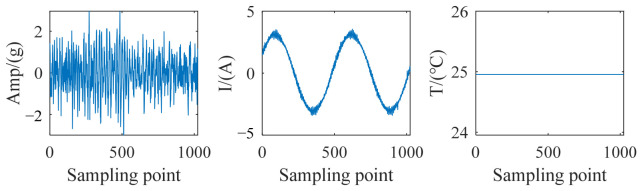
The raw vibration, current and temperature data under the healthy condition.

**Figure 7:**
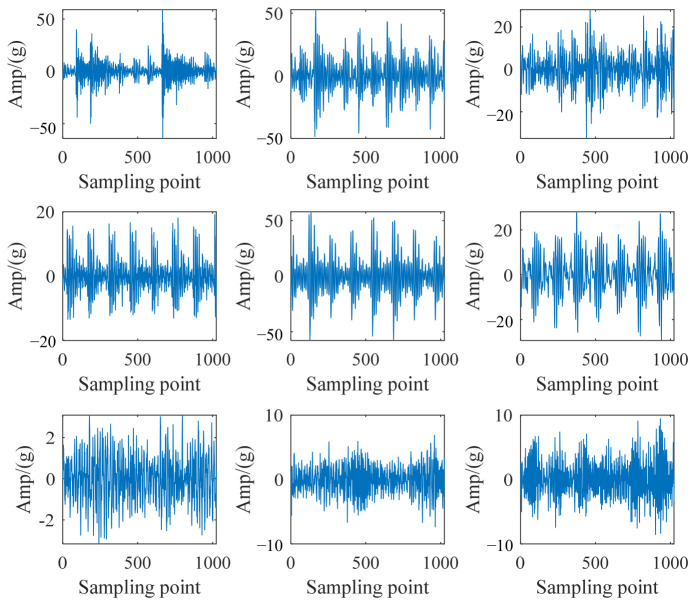
The raw vibration waveforms under the nine fault conditions.

For multi-sensor data under different operating conditions, a uniform sliding window strategy was adopted to extract time-frequency features, ensuring consistency and comparability of the input data for the model. Four vibration signals, one current signal and two temperature signals were selected for this purpose, with specific sample details provided in Table 1. The raw vibration, current and temperature data under the healthy condition are shown in Figure 6. As observed, the vibration amplitude remains largely below 2 g, the current exhibits a sinusoidal waveform with low noise, and the temperature stays constant at approximately 25 °C. Figure 7 depicts the raw vibration waveforms under the nine fault conditions. It can be seen that distinguishing fault types directly from the raw data is challenging, especially since the features within the same fault category appear highly similar.

**Figure 8:**
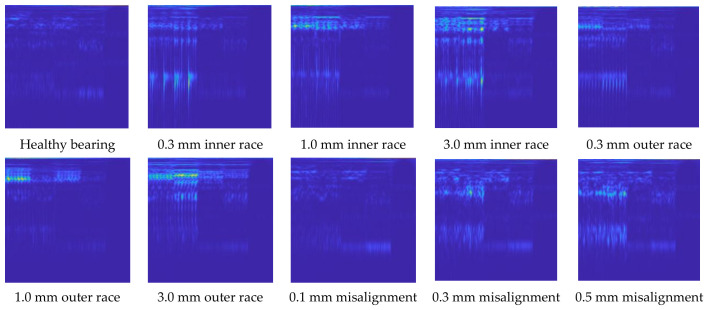
CWT-derived feature maps.

Thereafter, the vibration, current, and temperature signals collected from each sensor were fused. Subsequently, using the cmor3-3 as the mother wavelet, a wavelet transform was applied to the dataset to generate two-dimensional feature images. Figure 8 shows the CWT-derived feature maps obtained after fusing the four vibration signals and one current signal. Finally, the CNN-LSTM network was employed to classify the feature maps corresponding to the ten rotating machinery fault conditions.

### 4.3. Experimental Results Analysis

All experiments were conducted on a desktop computer equipped with an Intel(R) Core(TM) i5-1035G1 CPU @ 1.00GHz and 8 GB RAM (Santa Clara, CA, USA), running MATLAB R2023b. The deep learning models were implemented using the MATLAB Deep Learning Toolbox. The detailed parameter configuration of the CNN-BiLSTM model is in Table 2. To prevent overfitting and improve the generalization ability of the neural network model, dropout layers were applied after both the pooling layers and the fully connected layers. To assess the proposed model’s fault diagnosis performance on small-sample datasets, all image samples in the dataset were randomly shuffled. Then, the model was trained using the first 20 images from every 100-image batch, with the remaining 80 images serving as test samples. Given the random initialization of network weights, the model was run five times to ensure the reliability of experimental results and the mean accuracy was obtained.

#### 4.3.1. Impact of Multi-Sensor Fusion and Image Pixel Size

In fault classification with a CNN-BiLSTM network, the diagnosis performance depends on key parameters such as the sensor count and the image size after CWT transformation. Sensors of the same type installed at different locations can capture redundant information, while sensors of different types can obtain complementary information. This enables a comprehensive analysis of the target equipment’s condition, effectively improving the accuracy of fault diagnosis. The pixel size of an image dictates its information content and, consequently, affects the model’s required processing speed and architectural depth. Excessively small pixels may lead to insufficient information, missing features and loss of detail, thereby reducing diagnostic accuracy. Conversely, overly large pixels can slow down the diagnostic model and increase the complexity demands on deep networks. Table 3 provides details on the influence of specific sensor fusion strategies and pixel configurations on the accuracy and efficiency observed in this research.

As can be seen from the results in Table 3, multi-sensor fusion yields better performance than using a single-vibration sensor, and the higher the image resolution, the greater the accuracy of the prediction. For 128 × 128-pixel images, the prediction accuracy of four vibration signals is higher than that of one vibration signal. The average prediction accuracy is the same for the configuration of C and D, but the latter has a lower variance. The best performance is achieved with the configuration of E, which indicates that the complementary information contained in vibration, current, and temperature signals can be fully utilized. Although 128 × 128-pixel images yield the superior accuracy, they demand longer times for training and testing, substantially increasing computational costs, so it is not suitable for time-sensitive tasks. In contrast, 32 × 32-pixel images allow for faster processing, but their accuracy is somewhat compromised. Using 64 × 64-pixel images offer a better trade-off between computational cost and accuracy. The selection of image resolution should be tailored to the specific application. The findings indicate that a 64 × 64 resolution achieves an optimal balance, maintaining sufficient feature detail while managing computational demands. This resolution maintains the essential signal information with minimal impact on model training time and computational complexity. Consequently, this approach enhances the ability to process raw signals and perform fault classification efficiently. For sensor fusion at 64 × 64 resolution, the test accuracy of fusing four vibration sensors is higher than that of using only one vibration sensor. Adding one current sensor further improves test accuracy. Although adding two temperature sensors also increases test accuracy, the improvement is less significant compared to adding only one current sensor. This is because it is limited by the image pixels. Therefore, the preferred sensor configuration is four vibration signals plus one current signal.

#### 4.3.2. Model Diagnostic Results

**Figure 9:**
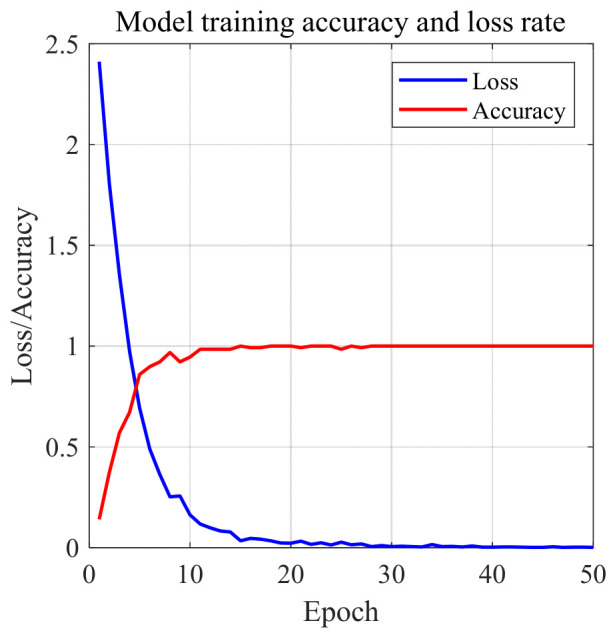
The training set accuracy and loss function values.

Figure 9 displays the training set accuracy and loss function values for the configuration fusing four vibration sensors with one current sensor, at a 64 × 64 resolution and a maximum of 50 iterations. As can be observed, the training accuracy exceeds 90% after only seven iterations and reaches 100% after 15 iterations. Regarding the loss function, it decreases to 2.41 after the first iteration. A progressive increase in the number of iterations results in a sharp decline in the loss value, falling below 0.1 after 12 iterations, below 0.01 after 28 iterations, and finally reaching 0.002. Combined with the data from Table 3, it can be concluded that the model training performs well without exhibiting overfitting.

**Figure 10:**
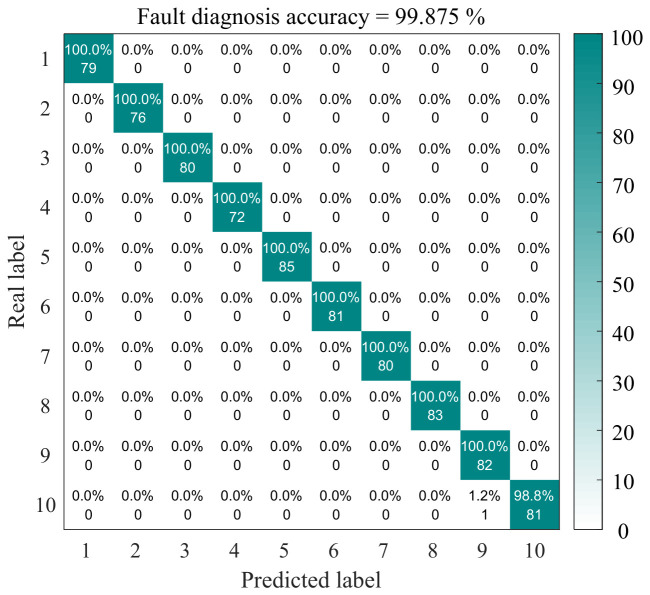
Confusion matrix for detailed analysis.

To more clearly demonstrate the recognition results of the model, Figure 10 presents a confusion matrix for detailed analysis, depicting the model’s recognition results for each test set category. Both its axes represent the ten different states. Except for one instance of a 0.5 mm misalignment fault being misclassified as a 0.3 mm misalignment fault, the remaining nine states were identified with 100% accuracy. These results demonstrate the robust fault identification capability of the CNN-BiLSTM model utilizing multi-sensor CWT image fusion, making it a viable tool for practical fault diagnosis in industrial applications.

### 4.4. Validation of Model Generalization Capability

#### 4.4.1. Analysis of Model Performance Under Varying Load Conditions

**Figure 11:**
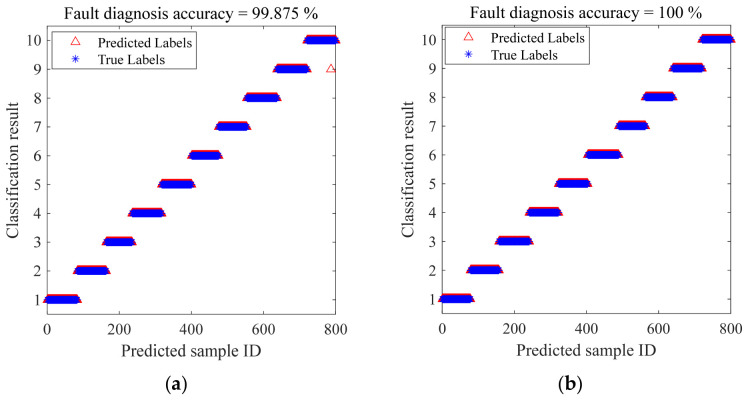
Predicted and true classification labels under 2 Nm and 4 Nm load conditions. (**a**) 2 Nm. (**b**) 4 Nm.

The preferred sensor fusion approach demonstrates excellent recognition performance on the aforementioned 0 Nm dataset. However, bearing loads in actual machinery can vary and significantly impact diagnostic accuracy. To this end, the classification performance of the proposed model was further evaluated under two additional load conditions (2 Nm and 4 Nm) using datasets of the same scale. Each scenario was validated independently, and visualization plots comparing predicted and true classification labels for all samples were generated, as shown in Figure 11. An accuracy of 99.88% was achieved by the diagnostic model in a single run under the 2 Nm load (detailed in Figure 11a). Under the 4 Nm load (Figure 11b), the model achieves 100% single-run accuracy. The plots comparing predicted and true classifications for all samples indicate that the model accurately identifies all 10 states across varying loads. This confirms that the method possesses both robustness and adaptability across different load conditions.

#### 4.4.2. Model Performance Analysis in Noisy Environments

The operational environment of equipment is typically complex and variable. Under actual working conditions, equipment is exposed to a range of influencing factors, including thermal variations, electromagnetic interference, and mechanical vibration. Sensor signals carrying these disturbances can degrade fault diagnosis results, making it crucial to evaluate the model’s noise resistance. To verify robustness under realistic industrial noise, Gaussian white noise was added to the signals. Specifically, six noise levels (SNR: −6, −4, −2, 2, 4, 6 dB), calculated via Equation (11), were introduced to the original data.

(11) $$SNR(dB)=10{log}_{10}(\frac{P_{signal}}{P_{noise}})$$

where $P_{noise}$ denotes the noise power, and $P_{signal}$ denotes the signal power.

**Figure 12:**
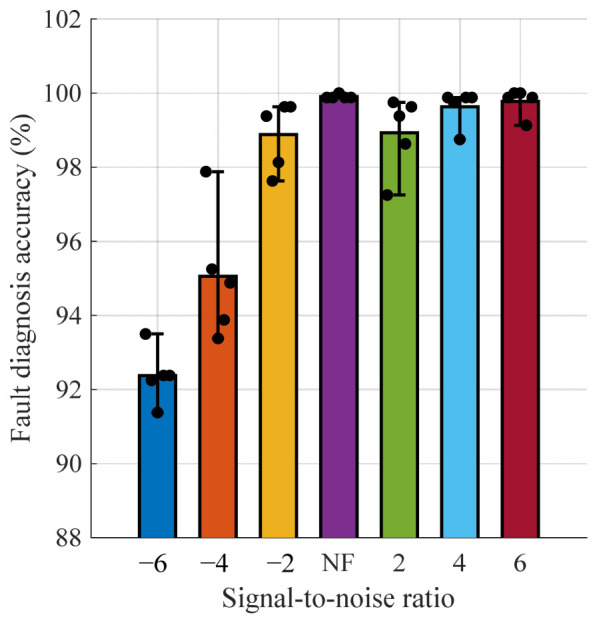
A comparative analysis of fault diagnosis at signal-to-noise ratios.

Subsequently, a comparative analysis of fault diagnosis was conducted between these noise-contaminated signals and the original noise-free data. The results are shown in Figure 12. Under ideal noise-free conditions, the model achieved near-perfect fault identification accuracy, approaching 100%. In a low-noise environment (SNR = 6 dB), the model exhibits robust performance, retaining an accuracy above 99.78% and confirming its reliability for typical industrial applications. With increased noise intensity, the diagnostic accuracy remains high at 99.93% even at an SNR of 2 dB. Even at the extreme −6 dB SNR, the model maintains an accuracy of 92.38% in identifying primary faults, attesting to its considerable robustness under high-noise conditions. This analysis of noise robustness validates that the model retains strong diagnostic capability under noisy conditions, demonstrating promising practical applicability.

It should be noted that although Gaussian white noise is a common benchmark for evaluating the robustness of models in noisy environments, the noise conditions in actual industrial environments are much more complex. Sources of interference in the real world include pulse noise generated by electrical switch operations or mechanical collisions, non-Gaussian noise resulting from unstable loads, as well as sensor drift caused by prolonged exposure to high temperatures. The impact of these non-Gaussian noises on the proposed CWT-CNN-BiLSTM framework still requires further investigation.

#### 4.4.3. Impact of Different Encoding Methods on Diagnostic Results

**Figure 13:**
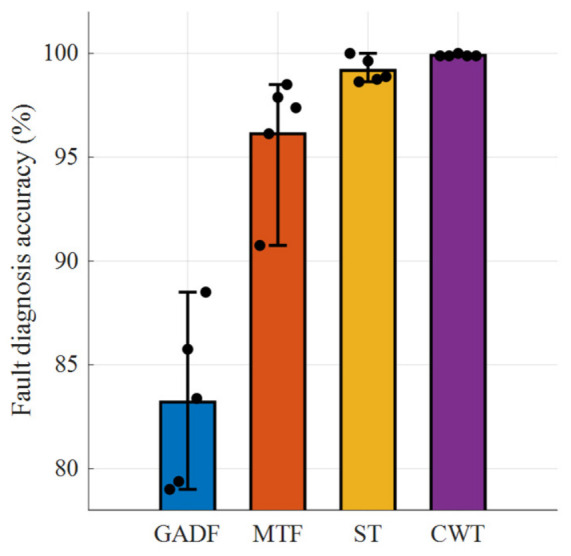
Impact of different encoding methods on diagnostic results.

This study benchmarks the CWT-CNN-BiLSTM model against several other image encoding techniques to further assess its effectiveness. During the experiments, the structure of the CNN-BiLSTM model remained unchanged, while the multi-sensor fusion data was converted into Markov Transition Field (MTF), Gramian Angular Difference Field (GADF), and S-Transform (ST) representations, respectively. The results are shown in Figure 13. The image encoding using CWT achieves the highest fault diagnosis accuracy. This is followed by the images encoded using the ST method, which attained an average fault diagnosis accuracy of 99.18%. It benefited from the advantages of ST that incorporates wavelet transform principles. The images encoded with GADF yields the lowest average fault diagnosis accuracy at 83.20%. However, it is still capable of effectively identifying the primary fault categories. These outcomes further underscore the clear advantages of the CWT-CNN-BiLSTM model.

#### 4.4.4. Impact of Different Network Algorithms on Diagnostic Results

**Figure 14:**
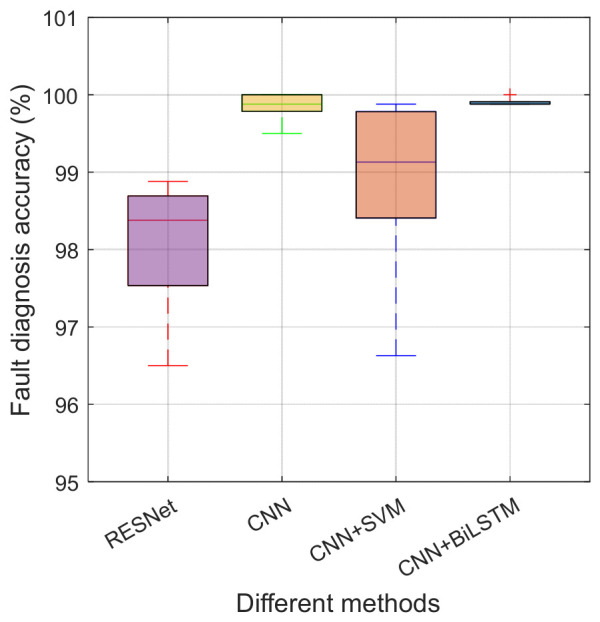
Impact of different network algorithms on diagnostic results.

As analyzed previously, images obtained by applying CWT to multi-sensor fused data provide richer fault information. However, whether the CNN-BiLSTM model holds an advantage over other models remains to be verified. This section continues this investigation. Using the multi-sensor fused CWT images as input, the diagnosis performances of RESNet, CNN [40], CNN-SVM and the proposed CNN-BiLSTM model were systematically compared. Boxplots are used to visually present the final experimental results. The results are shown in Figure 14. Overall, the diagnostic accuracy of all models is relatively close, each exceeding 98%, indicating the advantage of CNN in extracting spatial features. However, the CNN-BiLSTM model (99.90 ± 0.05%) slightly outperforms RESNet (98.05 ± 0.94%), CNN (99.85 ± 0.21%), and CNN-SVM (98.88 ± 1.31%). Moreover, the CNN-BiLSTM model has the smallest accuracy standard deviation, indicating superior result stability. This advantage is attributed to its effective integration of CNN’s spatial feature extraction and BiLSTM’s bidirectional temporal modeling capabilities, which yields significant diagnostic accuracy. Consequently, it provides a reliable and efficient fault diagnosis solution for multi-sensor fusion in rotating machinery system.

## 5. Conclusions

A small-sample fault diagnosis method is developed in this work, leveraging multi-sensor fusion and a CWT-CNN-BiLSTM model. On the basis of experimental comparison and analysis, the following conclusions are reached.

(1) The optimal configuration combines four vibration sensors and one current sensor, with CWT conversion to 64 × 64 resolution achieving the optimal trade-off between computational efficiency and diagnostic accuracy, ultimately yielding a 99.90% fault detection rate.
(2) To assess the model’s generalization, comparative tests are performed under diverse load and noise conditions. The results indicate that the model not only sustains superior diagnostic performance across load variations but also possesses robust noise resistance.
(3) Comparative analysis is conducted across different image encoding methods and network architectures. The results demonstrate that the CWT-based image encoding approach outperforms other methods in multi-sensor rotating machinery fault diagnosis. When evaluated against various network models, CNN-BiLSTM is found to achieve higher accuracy through deeper learning of CWT feature maps.

Despite the satisfactory results achieved, the current model still has some limitations. It relies on high-quality, synchronized multi-sensor data. Moreover, its effectiveness is currently limited to fixed-speed conditions and known fault patterns only. Our future work will expand the model’s applicability to cover more types of machinery and complex industrial operating conditions.

## Figures and Tables

**Table 1:** Ten state settings.

Sensor Type	Fault Type	Sample	Label
four vibration signals one current signal two temperature signals	healthy bearing	100	1
	inner race faults 0.3 mm	100	2
	inner race faults 1.0 mm	100	3
	inner race faults 3.0 mm	100	4
	outer race faults 0.3 mm	100	5
	outer race faults 1.0 mm	100	6
	outer race faults 3.0 mm	100	7
	misalignment faults 0.1 mm	100	8
	misalignment faults 0.3 mm	100	9
	misalignment faults 0.5 mm	100	10

**Table 2:** Parameter configuration of the CNN-BiLSTM model.

Network Layer	Input Size	Kernel Size	Stride	Padding	Output Size
Input	64 × 64 × 3	/	/	/	/
Conv1+BN1	64 × 64 × 3	3 × 3 × 16	1	1	64 × 64 × 16
Max_pool1	64 × 64 × 16	2 × 2	2	0	32 × 32 × 16
Conv2+BN2	32 × 32 × 16	3 × 3 × 32	1	1	32 × 32 × 32
Max_pool2	32 × 32 × 32	2 × 1	2	0	16 × 16 × 32
Conv3+BN3	16 × 16 × 32	3 × 3 × 64	1	1	16 × 16 × 64
Max_pool3	16 × 16 × 64	2 × 1	2	0	8 × 8× 64
Flatten	8 × 8 × 64	/	/	/	4096 × 1
BiLSTM	4096 × 1	128	/	/	256 × 1
FC1	256 × 1	64	/	/	64 × 1
FC2	64 × 1	32	/	/	32 × 1
FC3	32 × 1	10	/	/	10 × 1
Softmax	10 × 1	10	/	/	10

**Table 3:** Impact of multi-sensor fusion and image pixel size.

Image Pixel	Sensor Type	Training Time (s)	Testing Time (s)	Testing Accuracy
32 × 32 × 3	A	16.00 ± 1.45	0.36 ± 0.03	96.45 ± 0.56%
	B	15.42 ± 0.63	0.36 ± 0.04	99.03 ± 0.44%
	C	14.54 ± 0.40	0.35 ± 0.04	99.54 ± 0.62%
	D	15.26 ± 1.14	0.40 ± 0.01	99.75 ± 0.19%
	E	14.07 ± 0.83	0.36 ± 0.09	99.57 ± 0.30%
64 × 64 × 3	A	45.92 ± 4.84	0.80 ± 0.23	96.55 ± 1.17%
	B	43.65 ± 2.77	0.82 ± 0.12	99.10 ± 0.93%
	C	43.80 ± 2.58	0.76 ± 0.15	99.90 ± 0.05%
	D	51.90 ± 4.86	1.08 ± 0.48	99.80 ± 0.07%
	E	44.11 ± 1.23	0.79 ± 0.15	99.38 ± 0.59%
128 × 128 × 3	A	176.17 ± 8.15	3.11 ± 0.43	96.95% ± 1.14%
	B	173.28 ± 13.36	3.14 ± 0.60	99.25 ± 0.73%
	C	178.06 ± 9.51	2.92 ± 0.35	99.90 ± 0.16%
	D	178.80 ± 9.04	2.77 ± 0.18	99.90 ± 0.10%
	E	178.75 ± 18.60	3.01 ± 0.39	99.95 ± 0.07%

Notes: A: one vibration signal, B: four vibration signals, C: four vibration signals and one current signal, D: four vibration signals and two temperature signals, E: four vibration signals, one current signal and two temperature signals.

## Data Availability

The data presented in this study are available on request from the corresponding author because they involve core data and are not convenient for upload. If you have any requests, please contact the corresponding author. We appreciate your understanding.
